# Nanodroplet-Confined Electroplating Enables Submicron Printing of Metals and Oxide Ceramics

**DOI:** 10.1021/acsnano.6c01486

**Published:** 2026-06-10

**Authors:** Mirco Nydegger, Rebecca A. Gallivan, Arthur Barras, Henning Galinski, Ralph Spolenak

**Affiliations:** Laboratory for Nanometallurgy, Department of Materials, 27219 ETH Zürich, Vladimir-Prelog-Weg 5, 8093 Zürich, Switzerland

**Keywords:** microscale additive manufacturing, metal nanostructures, 3D nanofabrication, electrohydrodynamic ejection, EHD-RP

## Abstract

The fabrication of functional micro- and nanoelectronic devices requires the deposition of high-quality materials from different electronic material classes, such as conductors, semiconductors, and insulators. Establishing ultrahigh-resolution additive manufacturing as a viable addition to existing fabrication methods requires the combinatorial additive deposition of different electronic material classes. However, current techniques do not provide such a capability. Here, we demonstrate that droplet-confined electroplating, an ultrahigh-resolution additive manufacturing technique initially developed for metals as electrohydrodynamic redox printing (EHD-RP), allows the direct deposition of not only many metals but also metal oxides. Particularly, we demonstrate that applying fundamental electrochemical principles in combination with on-the-fly switching of the deposited material allows for the direct codeposition of metals, metal hydroxides, and metal oxides. Our results demonstrate the feasibility of leveraging simple water-based electrochemical concepts to produce intricate and multimaterial structures at the nanoscale.

## Graphical abstract

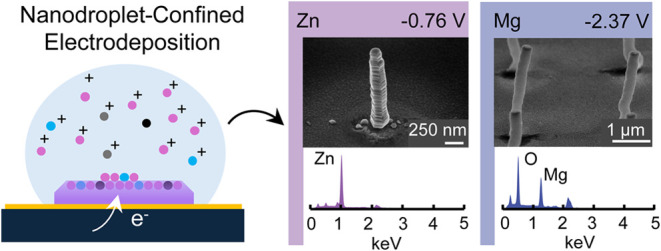

Electroplating is a long-established and foundational technique in materials science that enables the controlled deposition of metals and compounds onto conductive substrates through electrochemical reduction.^4^ The technique relies on an ionic exchange through a continuous electrolyte medium and is therefore traditionally performed in bulk electrolytic cells with well-defined anode–cathode configurations to deposit metal coatings. Now, miniaturized and localized versions of this process, such as meniscus-confined electroplating,^5,6^ are emerging as powerful tools for additive manufacturing at the micro- and nanoscale. In this confined geometry, a liquid bridge delivers the electrolyte to a targeted surface region, thus enabling the free-form deposition of metallic structures (Figure 1a).^7^ The approach of spatially confining the electrolyte has been pushed even further through electrohydrodynamic redox printing (EHD-RP), where electroplating is confined within isolated droplets of down to 50 nm in diameter (Figure 1b).^3^ The individual droplets, containing dissolved metal ions, are brought into contact with a conductive substrate by using electrohydrodynamic ejection from a nozzle.^1,8^ On the substrate, metal ions are deposited while the solvent evaporates. The result is a localized and transient electrochemical cell (Figure 1b) that evolves dynamically with the applied electric field and depends on the distance between the ejector and the substrate.^2^ The ejection frequency of the individual droplets is estimated to be ≈2.5 MHz^9^ and the current is in the range of 0.5–1.25 nA (which relates to a current density within the deposited structure of 1.5–4 A cm^–2^ for a pillar with 200 nm diameter). The change from a meniscus-confined liquid bridge to confinement within a droplet brings four major benefits: first, the forced mass transfer through a continuous stream of droplets allows a higher deposition speed than a diffusion-limited process would.^9–12^ Second, supplying droplets loaded with ions of different elements allows for a rapid change in the deposited metal,^1^ granting access to deposit structures with a chemical architecture. Third, the confined environment unlocks unique pathways for material synthesis, including access to metastable phases and nonequilibrium structures.^13^ Lastly, the deposition of individual droplets can be combined with an external source of electrons, such as an electron beam, to allow the deposition of metals also onto nonconductive substrates.^14^

**Figure 1:**
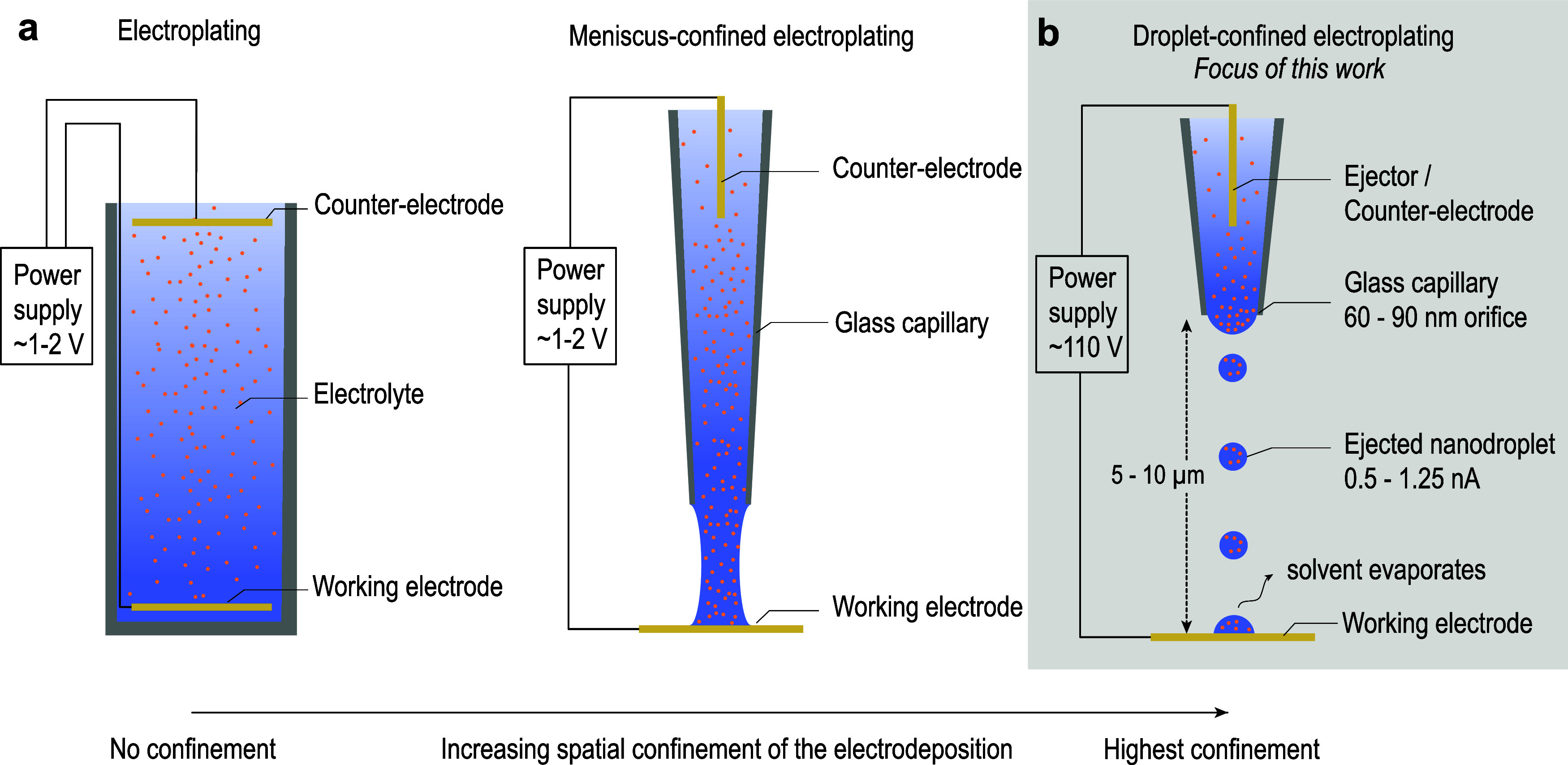
Two-electrode setup: from standard electroplating to droplet-confined electroplating. (a) In standard electroplating, the electrolyte is not spatially confined. Meniscus-confined electroplating confines the electrodeposition of metals into a small volume by using a liquid bridge between a solvent-filled capillary and a conductive substrate. This process is still very similar to normal electroplating, as both electrodes are connected through an electrolyte. (b) Droplet-confined electroplating is also based on a solvent-filled capillary in close proximity to a conductive substrate.^1^ Applying a high electric potential between an electrode immersed within the capillary and the conductive substrate leads to an electrohydrodynamic ejection of an ion-containing droplet. This droplet then impacts the substrate, the contained ions are reduced, and the droplet evaporates. A repetition of this cycle leads to the formation of an out-of-plane metal nanostructure. Notably, no liquid connection is maintained between the electrolyte reservoir and the individual droplets.^2,3^ .

Importantly, in droplet-confined electroplating, the electrolyte on the working electrode is no longer part of a continuous bath connected to a distant counter electrode.^2,3^ Instead, the spatial isolation introduces a fundamental change to the electrochemical environment: ionic exchange with a bulk reservoir is eliminated, and electrode reactions proceed under isolated conditions. As a result, traditional electroplating recipes presumably cannot be translated directly to the confined regime. This leads to a limited range of metals that have been reported so far (namely, only Cu, Ag, and Zn have been reported previously)^1,13,15^ but also to frequent observation of contaminants in the deposited structures.^13^ In addition, successful confined deposition requires suppression of electrostatic droplet instabilities, since excessive in-flight evaporation can drive the charged nanodroplets to the Rayleigh limit and lead to Coulomb explosions, resulting in spray-like rather than spatially confined deposition.^2,3^ Hence, the chemistry of the electrolyte must be controlled with the electrostatic stability of the ejected droplets in mind before droplet-confined electroplating can be used to create functional structures.

In the present study, we deposit a range of elements with standard electrode potentials between 1.83 V and −2.57 V. This systematic study enables us to derive a framework that specifically considers the role of speciation and chemistry of the ejected droplet in the deposition process. Such a framework may guide future research toward selecting ideal concentrations and chemistries for different elements. We then demonstrate that the established framework enables the deposition of materials with complex chemistry by utilizing the controlled codeposition of salts. Finally, we explore the deposition of composite structures—potentially opening simple pathways to additive manufacturing of functional metal/metal-oxide structures at the nanoscale.

## Results

### Droplet-Confined Deposition of Pure Metals

Previous works on droplet-confined electroplating focused on the deposition of Cu, Ag, and Zn.^1,13,15^ In the present study, we screen a larger variety of elements. We use 1 mM solutions of inorganic salts dissolved in water as electrolytes for the deposition of a selection of different elements (Figure 2). Usually, a chloride or sulfate salt has been selected (as described in the Experimental section). Each SE image of an element is accompanied by an EDX analysis of its chemical composition. The underlaid colors group elements by commonality in deposition behavior: For noble metals like Au, Pt, and Pd (shaded in yellow), the codeposition of chloride is observed. The deposits often do not form pillars or exhibit very slow growth rates. Importantly, the deposition time was not controlled between the screening experiments in Figure 2, so the apparent height of the Pt deposit should not be interpreted as evidence of faster pillar growth. For Au and Pt, deposits resemble previously reported results for low concentrations of precursor (≤0.1 mM).^13^ Presumably, for noble metals, the nominal precursor concentration of 1 mM does not correspond to the same effective concentration of fully solvated, electrochemically available metal species as for readily dissociating salts such as CuSO_4_, because chloroaurate remains strongly complexed and is transported together with chloride-containing ligands. Here, future work could investigate the possibility of removing Cl as a gas by providing a surplus of H^+^ during deposition. For Ag and Cu (shaded in pink), no deposition of the counterions is observed when deposited from 1 mM solutions (both Cl and SO_4_ salts have been used). For rhodium, a codeposition of the counterion is difficult to verify, as the Cl Kα line at 2.622 keV would overlap with the Rh Lα line at 2.696 keV. Nevertheless, a defined Rh structure was deposited, although with a high carbon content.

**Figure 2:**
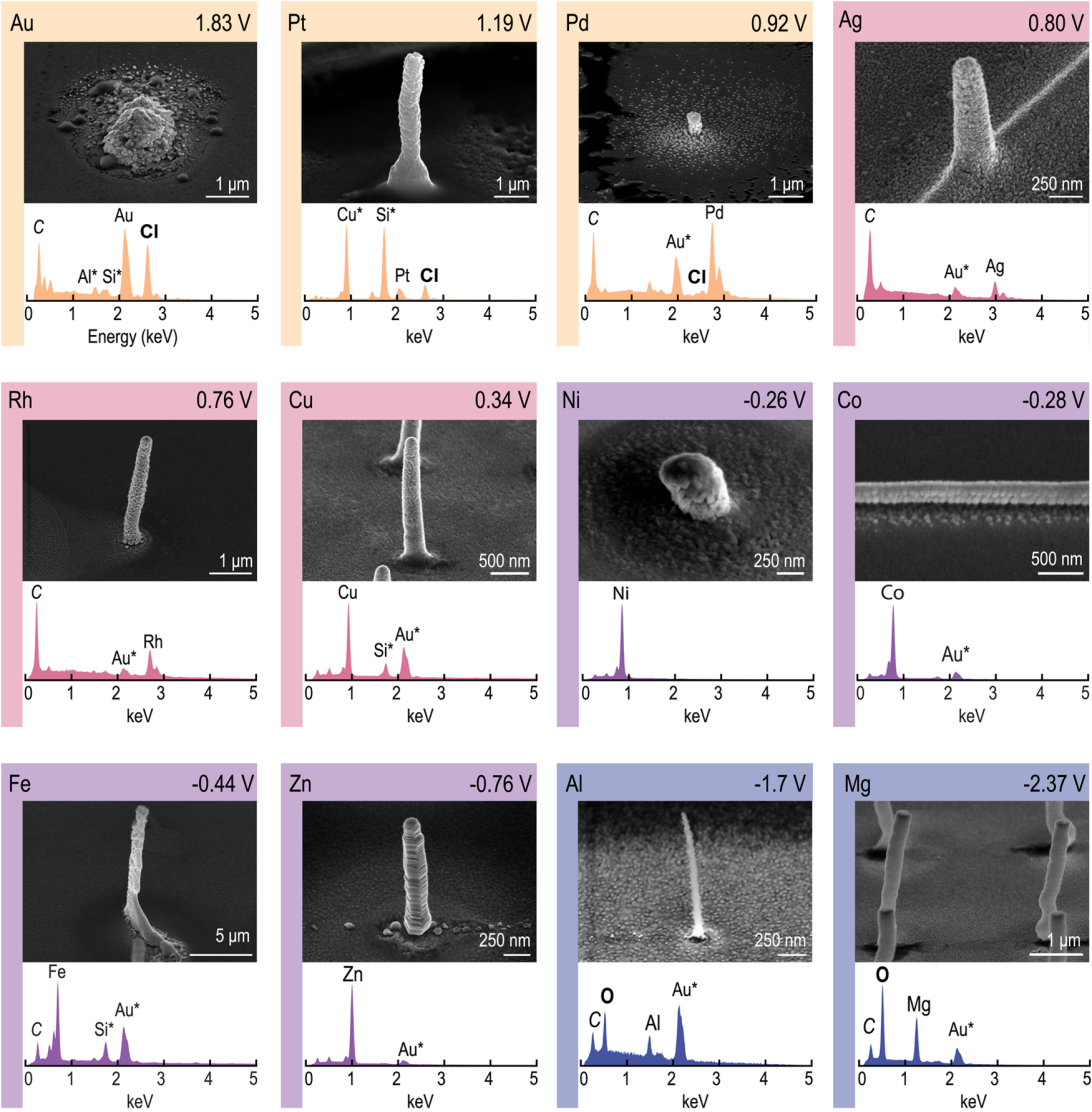
Extended range of elements available for deposition in droplet confinement. Scanning electron micrographs and EDX analyses of elements from a wide range of standard electrode potentials indicated in the top right of each box (relative to the standard hydrogen electrode, SHE). Elements marked with an asterisk refer to the used electrode metal (substrate material) and are not part of the deposited structure. Bold indicates noncarbonaceous elements present in significant quantities that are neither the deposited element nor the substrate. Elements shaded in yellow contained traces of counterions or complexing elements, namely chloride for the utilized chloride salts. Note that Au and Pt could only be ejected by applying a negative potential, presumably due to the negatively charged chloride ions. Elements shaded in red allowed for a facile deposition, while elements shaded in purple necessitated a pH between 3 and 5 to enable a deposition of a structure with low oxygen content. Elements shaded in blue always contained a significant amount of oxygen. The prevalence of carbon does not correlate with the electrode potential of the deposited material.

Elements with a standard electrode potential between 0 and −0.82 V (Ni, Co, Fe, and Zn, shaded in purple) required the addition of an acid (commonly HCl or HNO_3_ to a pH of 3–5) to enable the deposition of metallic structures.^15^ The influence of the pH is further illustrated in the Supporting Information (Figure S1), where pH-dependent EDX spectra are shown. Zn forms a distinctly faceted and highly crystalline whiskerlike structure, consistent with our previous observations for Zn. This morphology is most likely related to the comparatively high surface mobility of adsorbed Zn species during growth, and the terraced, faceted appearance arises from the growth of Zn under the directional addition of ions.^15^

It is known that elements with a standard electrode potential below the hydrogen evolution reaction at −0.82 V (Al and Mg, shaded in blue) cannot be electroplated in metallic form from an aqueous solution under normal conditions, as the water will be reduced preferably over the metal ions in solution. The cations will subsequently precipitate as hydroxides. Indeed, EDX analysis in Figure 2 shows high oxygen contents for Al and Mg pillars, as would be expected from hydroxides or oxides. The pillars appear as homogeneous white structures with no surface patterning and streaking of the image around the pillars’ periphery. These streaks are most probably due to charging of the structures under electron beam imaging and indicate poor conductivity. Dark-field transmission electron microscopy (TEM) reveals a highly porous structure (Figure 3). Electron diffraction indicates the presence of nanocrystalline MgO (note that because of the submicron dimensions and hence small interaction volume of the printed structures, XRD does not provide reliable phase identification). The ring patterns match well with the pattern expected for cubic MgO (space group *Fm*3̅*m*). High-resolution TEM confirms the presence of a nanocrystalline microstructure (Figure 3c).

**Figure 3:**
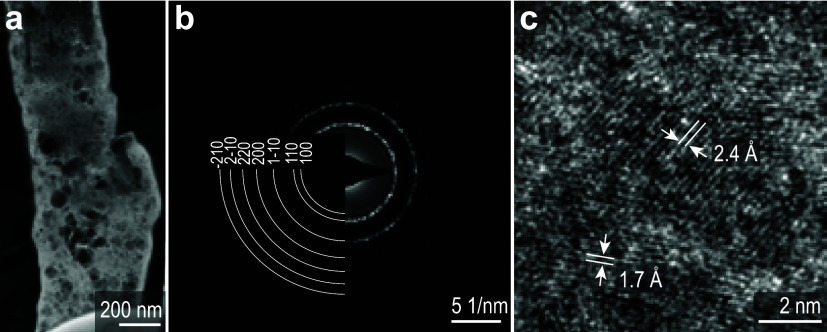
Analysis of deposited magnesium. (a) Dark-field TEM reveals a very porous microstructure of deposited magnesium compounds. (b) Selected area electron diffraction reveals a ringlike pattern that fits well with the expected pattern for nanocrystalline cubic MgO (space group *Fm*3̅*m*). (c) Presence of a nanocrystalline material further supported by high-resolution TEM.

### Toward Exotic Materials: The Curious Case of Nickel Phosphorus Oxide

The codeposition of counterions observed for noble metals here was also previously observed for CuSO_4_ at high concentration (≥10 mM).^13^ While codeposition is undesirable for the deposition of pure metals, it can be leveraged to allow for intentional incorporation of negatively charged species to create more chemically complex compounds. For example, introducing phosphorus ions by mixing a hypophosphorous acid (H_3_PO_2_) with a nickel salt solution results in codeposition of phosphorus species and the nickel salt anion (e.g., Cl^–^) into the printed Ni-based structures (Figure 4). While in previous work, high ion concentrations inhibited the formation of pillars and deposition yielded just planar deposits (which contained both metal and counterions), for this Ni–P–O system (Figure 4), well-resolved pillar structures are deposited. This is observed for similarly high ion concentrations, and the chloride- or phosphorus-containing anions are incorporated directly into the structures.

**Figure 4:**
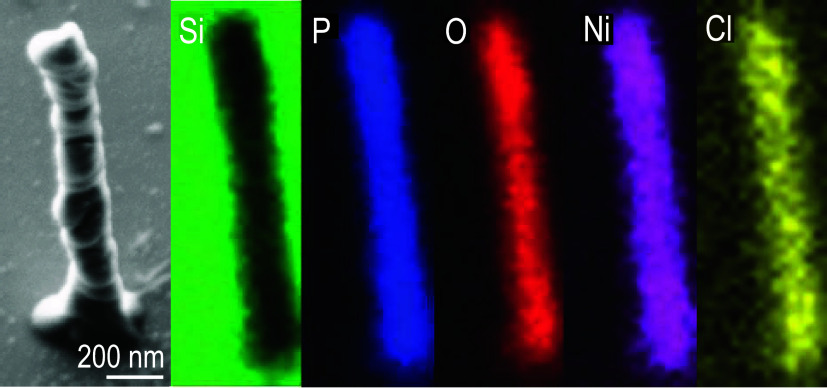
Deposition and chemical analysis of the Ni–P–O system. Deposited pillar from a mixture of an aqueous NiCl_2_ salt solution and an aqueous hypophosphoric acid solution (H_3_PO_2_) with EDS showing the presence of P (blue), O (red), Ni (pink), and Cl (yellow) in the final structure and the Si (green) of the substrate.

As noted above for Ni deposition, the pH must be low for the direct metal deposition. Although these solutions ranged from 2.55 to 2.75 in pH, depending on the Ni:P ratio, all printed structures contained strong oxygen signatures. This is in contrast to the pure NiCl_2_ solution, which produces pure Ni at a similar acidity. Because of the more complex deposition process with the added P and oxygen source from the hypophosphorous acid, this low pH may not be enough to inhibit hydroxide and oxide formation.

### Combining the Deposition of Different Material Classes

On the basis of promising initial results for the deposition of both metallic and oxidic monolithic structures, we extend the deposition to metal–ceramic composite structures. Here, we utilize two-channel nozzles as described previously,^1^ in which one channel is filled with 1 mM CuSO_4_ and the other with 1 mM MgCl_2_ (Figure 5a,b).

**Figure 5:**
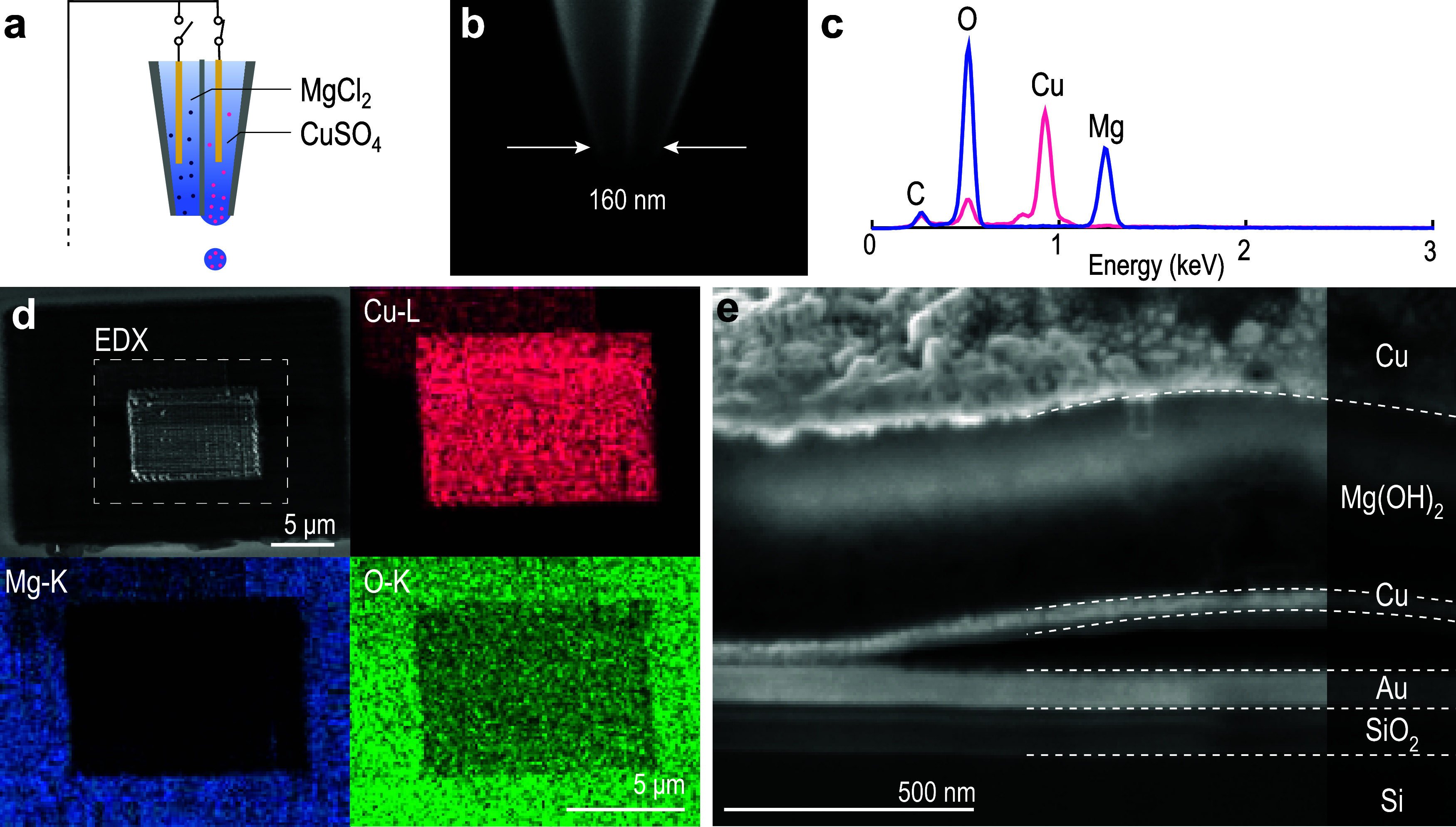
Codeposition of a metal (Cu) and a dielectric material (Mg­(OH)_2_). (a, b) Two-channel nozzle used for multimaterial-class deposition. One channel is filled with 1 mM CuSO_4_ and the other is filled with 1 mM MgCl_2_. Best deposition results were obtained for orifice sizes around 160 nm. (c) EDX analysis of separate Cu and Mg­(OH)_2_ pad indicates that the materials can be deposited separately from the same nozzle with little intermixing. Only a minor Cu peak can be observed in the Mg structure. (d) Low magnification image of a 5 by 5 μm Cu pad on top of a 10 by 10 μm magnesium hydroxide pad. The inset is shown in the EDX maps. The spatial distribution of Cu, Mg, and O indicates that Cu is only present in the square where it was deposited (the square at the top left in the Cu image originates from a shift in the position of the substrate during printing) and that Mg is mostly found in the larger pad surrounding the Cu. (e) Cross section of a Cu–Mg­(OH)_2_–Cu multilayered structure. The first Cu layer delaminated from the Au substrate. Both Cu layers appear much brighter than the porous Mg­(OH)_2_. Further, charging of the Mg­(OH)_2_ layer can be observed as a brighter section within the layer.

To verify independent deposition from each channel, structures were printed by using each channel separately. EDX analysis of the resulting deposits (Figure 5c) indicates that the two materials are largely deposited without intermixing, with only a minor Mg signal detected in the Cu region. More precise quantification of inadvertent cross contamination between channels would require higher-resolution techniques, such as electrospray mass ionization of ejected droplets^1^ or atom probe tomography.^3^ Figure 5d shows a SEM image depicting a Cu pad (brighter square in the middle) on top of a larger black square, which demonstrates that these structures can be printed on top of each other, hence effectively directly depositing multimaterial-class structures (i.e., metal and ceramics in the same structure). The magnesium oxide square is larger than the Cu pad to ensure that there is no connection between the Cu pad and the conductive Au substrate. EDX maps, taken of the smaller area depicted in Figure 5d, confirm that Cu is only deposited in the square pattern (the copper on top left is due to a shift of the nozzle position during printing). The larger black pad was confirmed to consist mainly of Mg and O. A cross section of a Cu–Mg­(OH)_2_–Cu pad (Figure 5e) also demonstrates that the layers can be stacked (although the bottom Cu layer delaminated from the Au substrate). It can also be seen that the Mg­(OH)_2_ layer is porous, and the top Cu layer exhibits a high roughness. While the Cu/Mg­(OH)_2_ structures shown here were obtained only within an initial process window, smoother metal layers and denser insulating layers should be achievable through optimization of deposition parameters such as deposition current and nozzle-to-substrate distance;^2^ if necessary, electron-beam-assisted deposition or irradiation could provide an additional route to improve layer quality.^14^

## Discussion

### Governing Electrochemical Principles in Droplet-Confined Electroplating

The elements and materials for which we demonstrated deposition are not a comprehensive list. We have therefore derived a framework to serve as a starting point for future work to deposit additional materials. The principles that govern the plating in droplet confinement are primarily due to the unique electrochemical setting of the technique, namely, the unidirectional ejection of cation-loaded droplets and the reduction within transient solvent droplets (i.e., all solvent evaporates intermittently or after the deposition), which prevents ion exchange with the larger electrolyte reservoir in the nozzle.^2,8^ The present work focuses on utilizing water as a solvent and electrolyte, although the principles found may apply analogously to other solvents. We use water as a model system here due to the extensive body of literature available for classical electroplating in water.^4,16^ However, the comparison to classical electroplating is complicated by the fact that the conditions (pH, metal ion concentration, temperature) within the droplets in EHD-RP are highly dynamic due to the simultaneous evaporation, reduction, and replenishment of electrolyte, while the nanoscopic size and the transient nature of the droplets render an experimental verification difficult. Therefore, we have to resort to more general statements that we base on both experimental observations and theoretical arguments. We discuss first the requirements for metal plating in the droplet and, based on these requirements, derive the requirements for the ion-generation mechanisms.

#### Reduction in the Droplet on the Substrate

##### Reactivity of the Metal

An obvious requirement for the deposition of a metallic structure is that the metal ion must preferably be reduced over the solvent on the substrate, which serves as the cathode. For water as a solvent, this information is already compiled as the standard electrode potential at standard conditions (25 °C, 1 M concentration for solutions, 1 atm pressure for gases). To deposit a metal from an aqueous solution, the reduction of the metal must take place preferentially over the cathodic partial reaction of water splitting (2H_2_O + 2 e^−^→ H_2_ + 2OH^–^; *E*°: −0.8277 V versus standard hydrogen electrode, SHE)^17^. A metal with a more negative electrode potential would lead to hydrogen formation and the precipitation of the metal as a hydroxide (M­(OH)_*x*_). This requirement is illustrated by the fact that metallic Zn (*E*°: −0.7618 V)^17^ was successfully deposited from aqueous acidic solutions, while magnesium and aluminum yielded oxygen-rich structures.

##### Counterions and Other Ligands in the Solvent Droplet

Since the droplet is not connected to a liquid reservoir that could allow for ion exchange, all nonvolatile species present in the droplet are ultimately deposited when the solvent evaporates. Therefore, we refer to all unwanted and nonvolatile species as contaminants. A notable source of contaminants is the counterions and other ligands of the metal cation. If, instead of cation-only loaded droplets, counterions are also ejected, partially reduced metal salts are deposited. This was previously observed for a 10 mM CuSO_4_ solution^13^ and in this work also for noble metals at 1 mM concentration (Figure 2).

We attribute this finding to the presence of metal salt complexes that are not fully dissociated. Such complexes can be detected by electrospray mass spectrometry (shown in previous work for Zn and NO_3_ complexes)^15^. In fact, speciation diagrams imply that many metals ions form complexes with counterions at concentrations of 1 mM or above.^18,19^ This point is particularly relevant for noble metal chlorides, which are known to form highly stable, partially covalent bonds.^20–22^ In fact, chloride was always detected (with EDX) in Au, Pd, and Pt structures deposited from the respective chloride salts at 1 mM concentration (Figure 2). Very low concentrations (≪1 mM) would have to be used to form fully solvated metal ions. Therefore, a second requirement for the deposition of pure metal structures can be determined: only fully solvated cations should be ejected.

Complexes with a net charge of zero (such as CuSO_4_) should not be attracted toward the nozzle apex by the electric field but are homogeneously distributed in the electrolyte and are therefore also coejected. Partially charged species, such as CuCl^+^, should also be attracted to the nozzle and are frequently coejected. Notably, the requirement that all cations should be fully solvated also excludes ligands that are typically used to improve the solubility of metal cations, such as ethylenediaminetetraacetic acid (EDTA). These chelating ligands usually form highly stable complexes, leading to the coejection of complexing agents.^23^

##### Toward Contamination-Free Metal Structures

Also, the third requirement for the deposition of pure metals emerges from the fact that all nonvolatile species present in the droplet are deposited. Nonvolatile species include unwanted positively charged ions (such as Na^+^ and Ca^2+^) but unfortunately also any uncharged nonvolatile species present in the electrolyte, which are not repulsed from the orifice by an applied electric field (like negatively charged counterions) and are therefore coejected. A high-purity deposited metal structure can be achieved by ensuring a high ratio of metal ions to other nonvolatile species in the droplet. In previous studies, metal-ion concentrations of 0.1 mM and below resulted in carbon-, sodium-, and calcium-rich deposits.^13^ The origin of the contamination has so far not been traced. Likely, the purity of the deposited metal structures is related to the ratio of metal ions to contaminants in the residing droplet on the substrate (Figure 6). The importance of this metal-to-contaminant ratio, therefore, naturally implies two possible directions of work toward purer metal structures: either the amount of carbon and other impurities has to be reduced or the amount of metal cations present in the droplet has to be increased.

**Figure 6:**
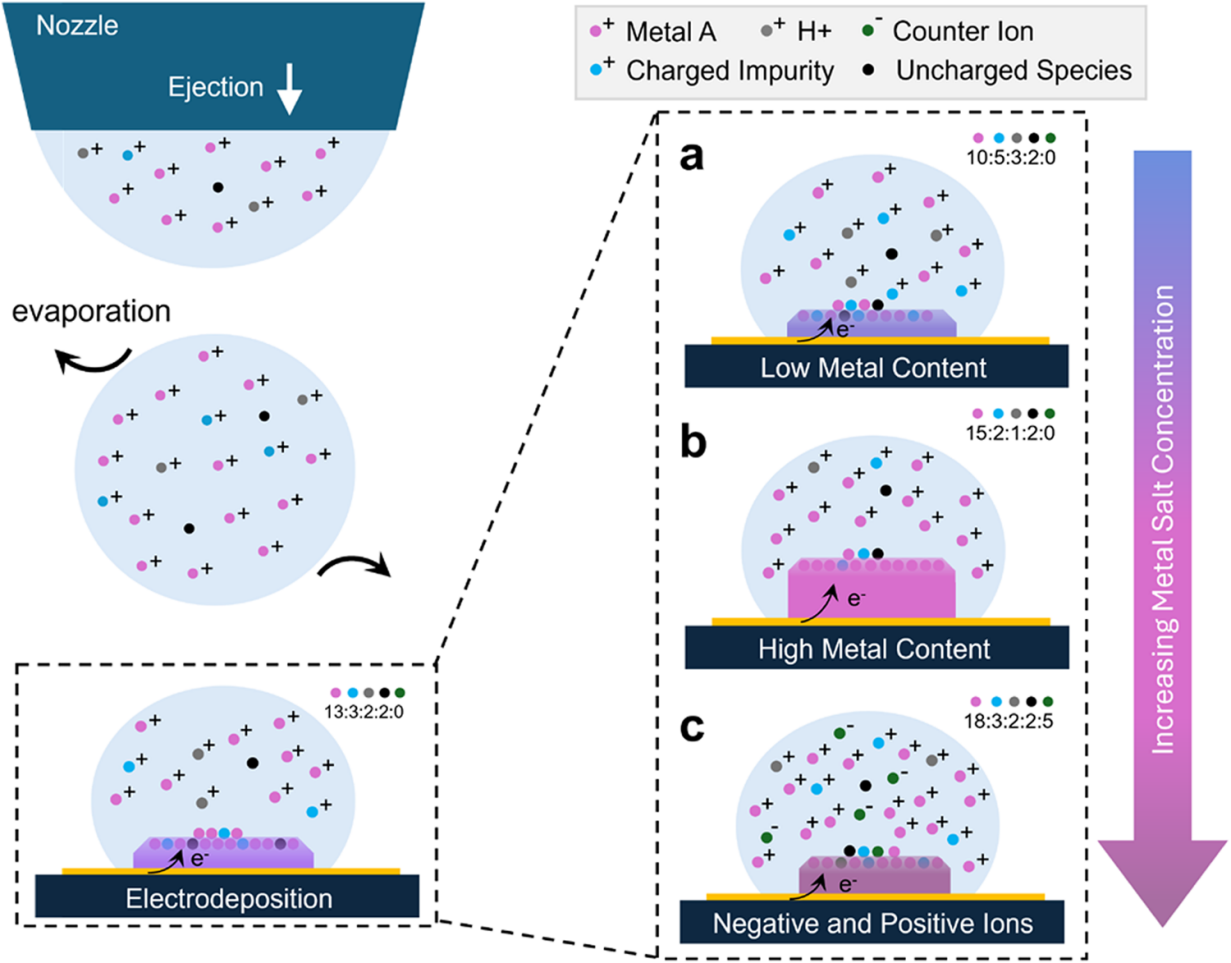
Importance of charge carriers on the purity of deposited structures. Schematic of ejected nanodroplets with a focus on charge carriers, including the depiction of dissolved metal A (pink), charged impurities (blue), H^+^ ions (gray), negatively charged counterions (green), and uncharged species (black). Note that the net charge of the droplet is conserved from ejection until it is reduced on the substrate or until the droplet disintegrates.^3^ The same net charge of the droplet can be achieved with various combinations of charge carriers. The specific ion counts shown in the schematics are illustrative only and do not represent experimentally or computationally validated droplet compositions; for an order-of-magnitude estimate of EHD-RP droplet charge, see the recent analysis of the radius-to-charge ratio in acetonitrile.^8^ (a) Low metal content and high content of H^+^ or charged impurities leading to a slow deposition of metal structures with large impurity incorporation. (b) High metal content and a minimal presence of H^+^ and charged impurities leading to the highest deposition rate. (c) At very high metal-ion concentrations, negatively charged counterions are coejected and subsequently codeposited. The counts of specific ions is to the right of each nanodroplet, and for all droplets, the net charge is equal.

First, the origin of carbon is currently not known but should be traced and avoided. So far, neither the use of an O_2_ plasma cleaner to clean all glassware before use nor ultrapure solvent has brought significant and reliable improvements in the observed levels of carbon in the structures. Here, an interesting approach could be to utilize electrospray mass spectroscopy to further investigate the nature of the carbon impurities with the goal of determining their origin.

Second, the finite charge budget of each droplet should be utilized as efficiently as possible for metal-ion transport. The total charge within a droplet is capped by the Rayleigh limit, an electrostatic stability threshold for charge-to-radius ratio that depends on the solvent properties, especially surface tension.^3^ Under a given charge budget, monovalent metal cations deliver more metal atoms per droplet than multivalent species and should therefore be preferred. Yet, many metals form bivalent species in water.^18,19^ Another contribution to the overall charge comes from the H^+^ ions present in the solution. However, the role of H^+^ is ambiguous. On the one hand, it could lead to porosity in the deposited structure due to hydrogen gas formation^2^ and reduce the amount of metal-cations per droplet due to its charge. The latter likely reduces the deposition efficiency (more droplets are necessary for the same volume of metal deposited), which, in combination with the assumption of a constant background level of contamination, results in less pure metal when printing at a lower pH. On the other hand, for ions like Ni and Fe, H^+^ may help improve the density by promoting the deposition of metals instead of hydroxides. One hypothesis is that the addition of H^+^ suppresses the local pH rise induced by water reduction in the transient droplet and thereby mitigates hydroxide precipitation. Here, after a local depletion in metal cations, the electrolysis of water leads to the formation of hydrogen gas and causes a local rise in the pH. This rise in pH then leads to the precipitation of metal hydroxides. The formation of hydroxides is a known phenomenon in electroplating, sometimes observed at locations of high current density (i.e., edges).^4,24^ For droplet-confined electroplating, the high current densities (0.6–1.8 A cm^–2^ ^2,9^ ) and a local electron surplus (provided by a capacitive effect of the setup) might lead to the onset of water electrolysis. Hence, H^+^ would enable the deposition of metallic structures for elements with an electrode potential between the cathodic partial reaction of water splitting and the reduction of hydrogen (−0.8277 V < *E*° < 0 V).^15^

#### Ion Generation in the Nozzle: Choosing Metal Salts and Electrode Materials

The observed contamination in the deposited structures implies a minimal cation concentration and therefore a minimal metal salt solubility of 0.1 mM in water, although the solubility is ideally above 1 mM for purer structures. The upper concentration limit for counterion-free deposition is likely specific to each salt. However, quantifying such an upper limit will require future studies that combine finer concentration sweeps with direct characterization of precursor speciation and codeposition behavior. Notably, however, the metal cations present can be from different elements. For example, a mixture of CuSO_4_ and ZnSO_4_ or Ag_2_SO_4_ leads to the deposition of an alloy with a stoichiometry equal to that of the utilized solution. Mixed salt solutions also allow for the fabrication of equiatomic Ag–Cu–Zn alloys. It is important for the deposition of alloys that compatible precursor salts be chosen. For example, a copper chloride precursor might be soluble, but the chloride anion would precipitate with any silver ions present due to the low solubility product of AgCl. Ideally, noncoordinating anions such as perchlorates are chosen.

Further, an immersed electrode is necessary to establish the electric field for the ejection and to regenerate the charges extracted at the tip. Without the formation of positive charges at the anode, the extraction of positive charges at the tip would lead to a net charge on the nozzle. This would then lead to an electric field counteracting the field used for droplet ejection and stop the ejection when the two contributions cancel each other out. Therefore, the anode should oxidize the solvent without passivating itself. To avoid the formation of secondary metal ions by anodic dissolution, a nondissolving, nonpassivating electrode should be used. The standard for droplet-confined electroplating is currently the use of an Au electrode when salt solutions are printed. The Au electrode allows for the oxygen-evolution reaction in the nozzle to ensure charge neutrality by the formation of H^+^ ions (*E*°: 1.229 V)^17^. Alternative nondissolving anodes with higher oxygen-evolution activity, such as Ir- or IrO_*x*_-based coatings on Au, could be an interesting future direction for achieving tighter control over proton generation and thereby the local droplet chemistry.

To summarize, the following set of requirements can be regarded as necessary for the deposition of pure metals in the confinement of transient water droplets:

(1) The concentration-adjusted standard electrode potential of the to-be-deposited metal is higher than −0.829 V.
(2) The metal ion forms a fully solvated species in the droplet.
(3) Concentration of metal cations in H_2_O ≥ 0.1 mM, given by the background level of contaminants and acidity (concentration of H^+^) in water.
(4) Mixed metal salts require sufficient solubility (≥0.1 mM) for all combinations of cations and anions.
(5) The electrode is made of a metal that has a standard electrode potential of >1.229 V.

### Direct Deposition of Insulators

The first open question for structures deposited from Mg and Al solutions concerns the actual nature of the deposited material—whether it is a metal, a hydroxide, or an oxide. We currently hypothesize that the difference between literature (predicting Mg­(OH)_2_)^25^ and electron diffraction (indicating MgO) is explained by a dehydroxylation of Mg­(OH)_2_ ^26^ when exposed to an electron beam (or high-intensity lasers) during analysis. Therefore, we assume that metal hydroxides are deposited; however, we note that further careful analysis of deposited structures is necessary.

The second open question is out-of-plane, insulating metal hydroxides can be deposited since the reduction of water on the substrate requires the presence of electrons. Here, one could entertain two possible reaction mechanisms: first, a water bridge connects the growth front to the conductive substrate, enabling the formation of hydroxide on the substrate, which later precipitates with incoming Mg cations. Second, and more probably, the charges can pass through the magnesium hydroxide itself. The very high applied electric fields, the porous nature of the hydroxide, and the arguably high level of contamination imply that the material does not act as a perfect insulator. Electron transfer through the deposited material would also explain the presence of metallic copper on top of deposited Mg­(OH)_2_, as shown in Figure 5. Interestingly, the deposited material appears as nonconductive under the electron beam (indicated by the horizontal streaks in many SEM images) in Figure 5. This could be related to different strengths of electric fields during deposition and analysis or that the low current during deposition could tunnel through the insulator (the deposition utilized a current of 1 nA, while SEM was carried out with a current of 0.1 nA; however, the e-beam is much more focused than deposition; hence, the deposition had a lower current density).

Future experiments should determine the conductivity or the breakdown electric fields of the deposited Mg­(OH)_2_, as this information is crucial to determining the reaction path. Further, UV–photoluminescence^27^ could give information about the insulating behavior of the deposited material (although care has to be taken to avoid an annealing of the material with the laser beam), as this technique is, in principle, sensitive to electronic states of defects within the band gap. Finally, an experiment determining the maximal height of the printed Mg­(OH)_2_ structures would be important. Future developments could use a dehydroxylation under irradiation to optimize deposition toward dense oxides. Here, one could combine the deposition of Mg­(OH)_2_ with in situ annealing using an electron beam or a focused laser beam, as has been used in previous studies to heat the substrate locally.^14,28^

### Case Study for Ni–P–O

On the basis of the findings for metallic and insulator deposition, the Ni–P–O system is presented as a case study to explore the deposition of functional materials with increased chemical complexity. Specifically, doped transition-metal oxides, such as phosphorus-doped NiO_*x*_, represent a class of tunable wide-band gap semiconductors with promising applications in photonics^29,30^ and integrated electronics.^31,32^ The results from the Toward Exotic Materials: The Curious Case of Nickel Phosphorus Oxide section already highlight the versatility of nanodroplet-confined electrodeposition, as Ni–P–O material systems are not typically produced through electrochemical methods. Furthermore, electrodeposition of Ni–P alloys often involves a two-step process at elevated bath temperatures as discussed in the Supporting Information. These factors point to the highly kinetic nature of deposition in these confined nanodroplets and a new opportunity for the design of chemically complex materials.

However, challenges remain with introducing dopants such as P, which generally form negatively charged complexes (e.g., PO^2–^), without incorporating the metal salt’s cation (Figure 4). To circumvent this issue, Ni^2+^ ions are introduced through electrocorrosion of a pure Ni wire in an aqueous hypophosphorous acid solution. (Details on selection of hypophosphorous acid over phosphoric acid are outlined in the Supporting Information.) This produces Cl-free materials that contain only Ni, P, and O, as shown in Figure 7.

**Figure 7:**
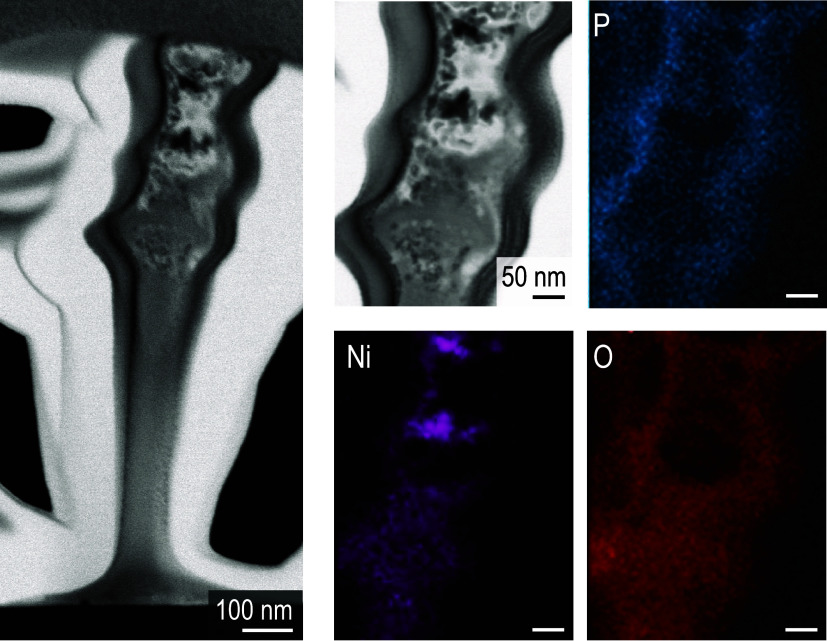
Dark-field transmission SEM image of a deposited pillar. The pillar is deposited from an aqueous hypophosphorous acid (H_3_PO_2_) solution containing Ni^2+^ ions generated by anodic dissolution of a Ni wire. Zoomed-in section of pillar top shows EDS mapping of P (blue), Ni (pink), and O (red).

Both metal salt and corrosive wire ion sources produced pillars with a strong oxygen signal and a clear presence of incorporated P. These structures also exhibit globular surface features, suggesting a greater fluctuation in droplet-to-droplet deposition compared with the pure chemistry of Ni salts (Figure 2). Transmission SEM images of a cross section of a Ni–P–O pillar highlight the strong presence of oxygen throughout the structure. It reveals limited crystallite formation near the base, although some larger Ni-rich grains were formed near the top (Figure 7). This points to the deposition of amorphous phosphorus-containing nickel hydroxide. Such hydroxides are observed in Ni-salt-based deposition, as discussed previously. While the pH of the solution in these depositions is below the observed threshold of 3–5, other kinetic effects, such as the presence of P or additional reactions at the surface, may shift the propensity for hydroxide formation. These deposited Ni–P–O structures also show some Ni-rich crystallites that have formed within the amorphous matrix. While the observed Ni-rich crystallites may form during deposition, beam-induced crystallization during either imaging or ion milling cannot be discounted.

Furthermore, the time-resolved Raman spectra in Figure 8 contain a broad initial peak with two sharper peaks at 1339 and 1585 cm^–1^. The broad peak, which disappears within the first 10 s of data collection, could be attributed to an amorphous phase such as an amorphous nickel hydroxide Ni­(OH)_2_, which is then annealed under the laser. The Raman spectra after 10 s appear similar to other spectra observed in carbon-containing Ni_3_(PO_4_)_2_.^33^ These peaks remain stable over 25 min of exposure (Figure 8). Raman peaks between 1220 and 1360 cm^–1^ are associated with P–O bonds^34^ while the second peak at 1585 cm^–1^ is aligned with a nanocrystalline NiO_*x*_ containing high defect densities.^35^ This peak aligns well with the NiO_*x*_ 2 M peak, indicating magnetic ordering. However, the effects of P on peak position and shape are not well understood and likely contribute to a slight deviation in peak position. Coupled with the EDS spectra and microscopy images, these results further point to an initial amorphous Ni­(OH)_2_ structure, which is likely annealed under the Raman laser and reduced to a stable Ni–P–O system.

**Figure 8:**
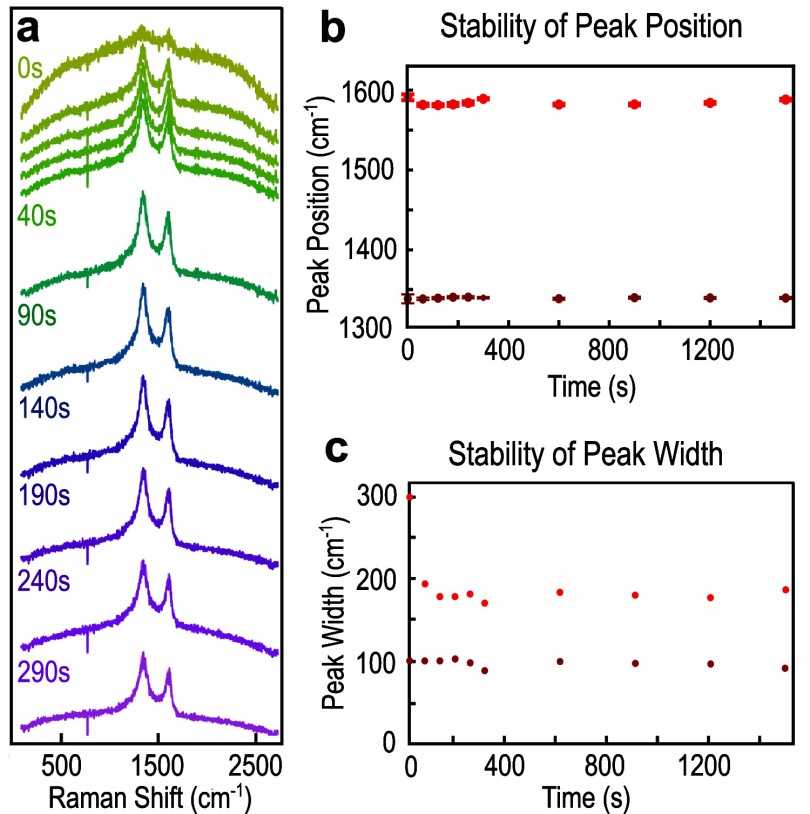
Stability of Ni–P–O under laser illumination. (a) Raman spectra offset to highlight different timesteps in the exposure with 0–40 s in 10 s intervals and 40–340 s in 50 s intervals. Dark yellow is the initial time step, and the bright purple is the last time step. (b) Peak position of both visible peaks over the full 25 min of exposure and (c) width of those peaks over the exposure.

This case study highlights the opportunity to design unusual, chemically complex materials at the nanoscale through confined nanodroplet deposition. By leveraging the presence of counterions or negatively charged species, this technique can introduce doping or advanced alloying to materials from ceramics to metals and semiconductors. This Ni–P–O system also highlights the opportunity to mix solution-based ions with ions introduced through electrocorrosion. Such methods are critical when needed to select a specific negatively charged ion species and could be extended to control metal-ion incorporation through electrocorrosion.

## Conclusions and Outlook

In summary, we have demonstrated that EHD-RP can be used to additively manufacture a much wider range of metals with submicrometer resolution than previously reported. Specifically, we investigated different approaches to provide a sufficient cation concentration in the electrolyte. Here, the use of metal salt solutions and diluted acids provided access to Co, Ni, Au, Pd, and Rh structures in addition to the previously reported Zn, Cu, and Ag structures.

To guide future searches for printable metals, we have attempted to derive the fundamental principles, governing deposition in transient solvent droplets. A set of five rules has been derived, based on both fundamental arguments and experimental results.

However, before reliable deposition of functional structures can be attempted, both the purity of the deposits and the reliability of the process must be increased. A major challenge appears to be the optimization of the ratio of ligand-free metal ions to other nonvolatile species. Therefore, future work should focus not only on tracking the source of contamination but also on investigating the role of H^+^ and testing other salts with that form less complexes, such as perchlorates.

## Experimental Section

### Materials

Nozzles for deposition were fabricated on a P-2000 micropipette puller system (Sutter Instruments) from filamented quartz capillaries (Sutter Instruments, Item QF100-70-15). Nozzle diameters were determined on a Quanta 200F (Thermo Fisher Scientific), equipped with a Schottky-type field-emission gun (FEG) in low vacuum mode (30 Pa) to avoid charging. Nozzles with diameters of 60–90 nm for a single channel and 160 nm for a dual channel were used. The nozzles were cleaned on the inside by rinsing with the solvent. Substrates were 0.4 cm × 2.0 cm pieces of Au-coated (80 nm) Si wafers. The substrates were cleaned in technical acetone, analytical isopropanol, and subsequently blow-dried with compressed air before use. Au wires (0.25 mm Metaux Precieux SA, 99.999%) were used as ejector electrodes. The wires were etched in concentrated nitric acid (65% HNO_3_, Sigma-Aldrich) for 30 s, dipped in water, and stored in air.

For the preparation of aqueous metal salt solutions, high-purity water (LC/MS-grade, Fisher Chemical) and the following metal salts were used: CuSO_4_·5H_2_O (Sigma-Aldrich, 99.999% metal basis), CuCl_2_·2H_2_O (Sigma-Aldrich, 99.999% metal basis), Ag_2_SO_4_ (Alfa Aesar, 99.999% metal basis), ZnCl_2_ (Alfa Aesar, 99.995% metal basis), CoCl_2_·6H_2_O (Sigma-Aldrich), HAuCl_4_·3H_2_O (Sigma-Aldrich, ≥49.0% metal basis), PdCl_2_ (Fluorochem Ltd.), RhCl_3_·4H_2_O (99.99% metals basis, Alfa Aesar), FeSO_4_·7H_2_O (Fluka Analytical), NiCl_2_ (Sigma-Aldrich, 99.999% metal basis), and PtCl_6_H_2_·6H_2_O (Sigma-Aldrich, ≥37.5% metal basis). All chemicals were used as received. The nozzles were filled by using gastight glass syringes with custom-made PEEK tips.

For the salt-based ion source in the Ni–P–O experiments, an aqueous solution with a 10 mM total ion concentration was mixed using a 2:3 Ni:P ratio by using NiCl_2_ (Sigma-Aldrich, 99.999% metal basis), hypophosphorous acid (H_3_PO_2_; Sigma-Aldrich), and high-purity water (LC/MS-grade, Fisher Chemical). For the wire corrosion ion source in the Ni–P–O experiments, ions were produced from a Ni wire (Alfa Aesar, Premion, 99.999% metal basis) in a 10 mM hypophosphorous acid (H_3_PO_2_, Sigma-Aldrich) aqueous solution made using high-purity water (LC/MS-grade, Fisher Chemical).

### Deposition

Deposition was performed at room temperature in an argon atmosphere (<100 ppm of O_2_). Typical potentials applied to the anode during printing were 80–160 V. The nozzle-substrate distance was controlled to 5–15 μm by analyzing light microscope images and adjusting the position of the *z*-axis piezo stage accordingly.

### Analysis

High-resolution scanning electron microscopy (SEM) was performed with a Magellan 400 SEM (Thermo Fisher Scientific, former FEI) equipped with an Octane Super EDX system (EDAX, software: TEAM). Tilt angles were 55°. HR-SEM images were taken in immersion mode at an acceleration voltage of 5 kV. A dual-beam Helios 5UX (Thermo Fisher Scientific) with a focused Ga^+^ liquid-metal-ion source was used for focused-ion-beam (FIB) milling of cross sections. Prior to FIB milling, the pillar was coated by a protective carbon layer, which is visible in the dark-field TEM image as a light gray background. Transmission mode SEM imaging and EDS were also performed in the dual-beam Helios 5UX.

## Supplementary Material



## Notes

Description of the setup used; EDX analysis of Ni and Fe printed at different pH values; electrochemical reactions of components in bath for Ni–P plating (PDF)

## ‡

Department of Aeronautics and Astronautics, MIT, 77 Massachusetts Avenue, Cambridge, Massachusetts 02139, United States

## §

Thayer School of Engineering at Dartmouth College, 15 Thayer Drive, Hanover, New Hampshire 03755, United States
