# Supplementary material for: Nanodroplet-Confined Electroplating Enables Submicron Printing of Metals and Oxide Ceramics
Source: ACS Nano. 2026 Jun 10;20(24):17316–26. doi: 10.1021/acsnano.6c01486 (PMC13296607; doi:10.1021/acsnano.6c01486)
Supplement: Supplementary file 1 [file nn6c01486_si_001.pdf]

Supporting Information

Nanodroplet-Confined Electroplating Enables Submicron Printing of Metals and Oxide Ceramics

Mirco Nydegger[1,2†], Rebecca A. Gallivan[1,3,†*], Arthur Barras[1], Henning Galinski[1] and Ralph Spolenak[1,*]

[1]Laboratory for Nanometallurgy, Department of Materials, ETH Zürich, Vladimir-Prelog-Weg 5, 8093 Zürich , Switzerland

[2]Present address: Department of Aeronautics and Astronautics, MIT, 77 Massachusetts Avenue, Cambridge, MA 02139, USA

[3]Present address: Thayer School of Engineering, Dartmouth College, 15 Thayer Drive, Hanover, NH 03755, USA

[†]These authors contributed equally

[*]To whom correspondence should be addressed; e-mail: rebecca.gallivan@dartmouth.edu; ralph.spolenak@mat.ethz.ch

1 Setup

Fundamentally, the setup consists of piezo stages that move the substrate in the X, Y, and Z directions (QNPXY-500, QNP50Z-250, Ensemble QL controller, Aerotech). Additionally, stage translations in X and Y directions larger than 500 µm were enabled by additional long-range stages (M112-1VG, PI for Y direction, manual micrometre screw, Mitutoyo for X direction). Piezo stages and power source were controlled through a custom Matlab script. The nozzle is mounted on a motorized nozzle holder (Z825B, controlled with Kinesis, both Thorlabs). The deposition is observed through an optical microscope composed of a $\times50$ objective lens (LMPLFLN, Olympus) and a CMOS camera (DCC1545M, Thorlabs). The lens is mounted at an inclination of 60 °to the substrate normal and the substrate is illuminated from behind using a green LED (LEDMT1E, Thorlabs). The resolution limit of the optical system is 530 nm, based on the green light ($\lambda = 530\ nm$) and an NA of 0.5. A power source (B2902a or B2962a, Keysight) with triaxial cable connectors was used for polarizing the anodes. Up to 4 channels can individually be supplied with high potential through a custom-designed PCB board, which utilizes reed-relays to open and close electrical connections (PICKERING, 131-1-A-5/1D). The metal wires were connected using a mechanical clamp. The complete printing setup is mounted inside a custom-built gas-tight box to provide a low-oxygen atmosphere (controlled with an oxygen sensor, Module ISM-3, Dansensor) and is placed on a damped SmartTable (Newport) to provide a vibration-isolated environment.

2 pH-dependent EDX

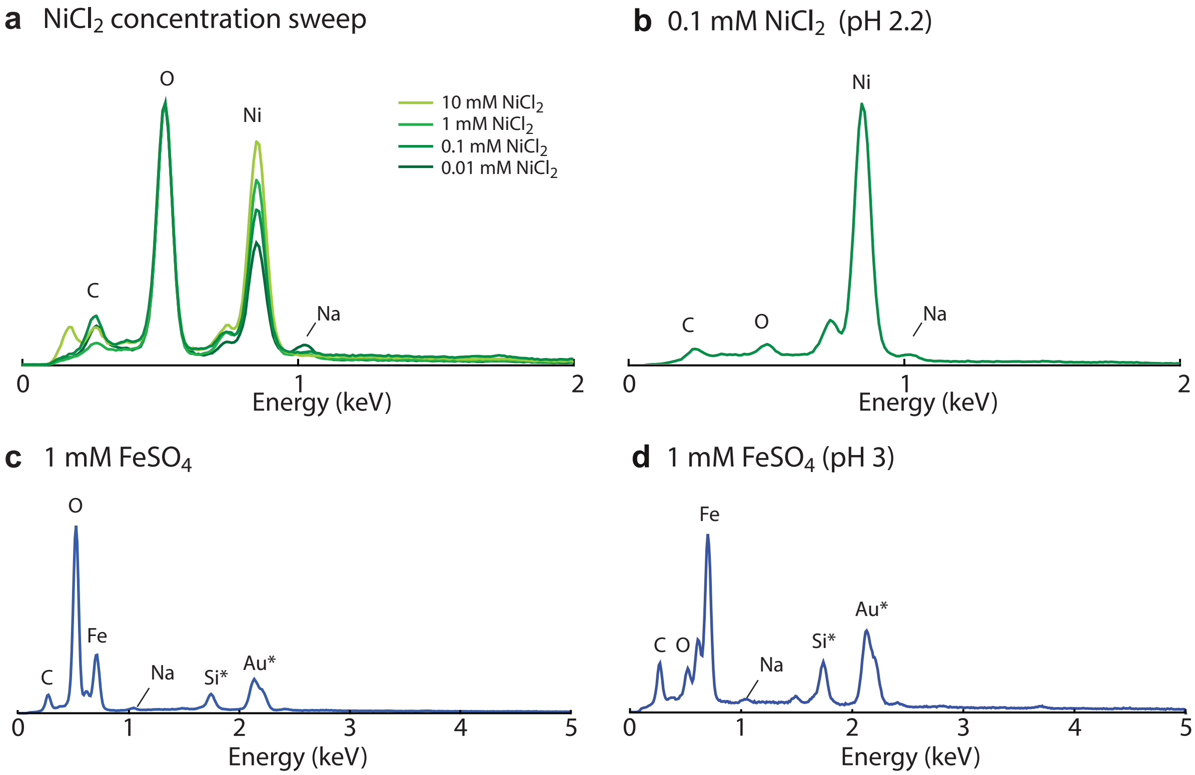

Figure S1: Influence of the concentration and the pH on deposited material. (a) A higher Ni concentration leads to a higher Ni to O ratio. **(b)** A pH of 2.2 suppresses the oxygen signal almost completely. **(c-d)** For Fe, a similar reduction of the O peak can be observed with a low pH in the electrolyte.

The O-to-Ni ratio depends on the $NiCl_2$ concentration in the electrolyte (Appendix Fig. S1a). A higher $NiCl_2$ concentration led to a lower O-to-Ni ratio (however, at 10 mM a Cl signal was found in the EDX analysis). The lowest O content was found when the pH was lowered to 2.2. With such a low pH, very slow deposition of structures was observed (20–30 s for the structure depicted in Fig. 2b), but EDX analysis revealed a much lower oxygen content and the surface shows a different morphology. Similar results to Ni were obtained for Fe, which was deposited from 1 mM $FeSO_4$ solutions. Here, for an unadjusted pH, structures with high oxygen content were deposited, while the addition of sulfuric acid (pH 3) enabled the deposition of Fe with a lower oxygen content (Appendix Fig. S1c-d).

3 Choice of Acid for the deposition of the Ni-P-O system

Ni-P compounds have previously been electrodeposited from a water-based electrolyte solution using either phosphoric acid (H_3PO_4) or hypophosphorous acid (H_3PO_2)[1]. The deposition of Ni-P can occur via either a direct or indirect mechanism of reducing P from a phosphorus-containing acid[2]. Table S1 highlights both routes in the half reactions that precede Ni-P electrodeposition.

Electrochemical reactions of components in bath for Ni-P plating

$$2H^+ + 2e^- \rightleftharpoons H_2 \quad (1)$$
$$2SO_4^{2-} + 4H^+ + 2e^- \rightleftharpoons S_2O_6^{2-} + 2H_2O \quad (2)$$
$$Ni^{2+} + 2e^- \rightleftharpoons Ni \quad (3)$$
$$H_3PO_4 + 2H^+ + 2e^- \rightleftharpoons H_3PO_3 + H_2O \quad (4)$$
$$H_3PO_3 + 3H^+ + 3e^- \rightleftharpoons P + 3H_2O \quad (5)$$
$$H_3PO_3 + 2H^+ + 2e^- \rightleftharpoons H_3PO_2 + H_2O \quad (6)$$
$$H_3PO_2 + H^+ + e^- \rightleftharpoons P + 2H_2O \quad (7)$$
$$P + 3H_2O + 3e^- \rightleftharpoons PH_3 + 3OH^- \quad (8)$$
$$SO_4^{2-} + H_2O + 2e^- \rightleftharpoons SO_3^{2-} + 2OH^- \quad (9)$$
$$PO_4^{3-} + 2H_2O + 2e^- \rightleftharpoons HPO_3^{2-} + 3OH^- \quad (10)$$
$$HPO_3^{2-} + 2H_2O + 3e^- \rightleftharpoons P + 5OH^- \quad (11)$$
$$SO_4^{2-} + 4H^+ + 2e^- \rightleftharpoons H_2SO_3 + H_2O \quad (12)$$
$$H_2SO_3 + 4H^+ + 4e^- \rightleftharpoons S + 3H_2O \quad (13)$$
$$Cl_2 + 2e^- \rightleftharpoons Cl^- \quad (14)$$

Table S1: Table showing selected half-reactions relevant to Ni-P electrodeposition, as outlined by Daly and Barry[1].

Given the time-sensitive and kinetically driven nature of the EHD-RP process, the direct mechanism of depositing solid P is far more promising. Thus, H_3PO_2 was selected for these experiments as it can be directly reduced to elemental phosphorus in an acidic environment.

References

[1] Daly, B. P.; Barry, F. J. Electrochemical nickel–phosphorus alloy formation. *International Materials Reviews* **2003**, *48*, 326–338.

[2] Pasek, M. A.; Sampson, J. M.; Atlas, Z. Redox chemistry in the phosphorus biogeochemical cycle. *Proceedings of the National Academy of Sciences* **2014**, *111*, 15468–15473.
